# Influence of Arbuscular Mycorrhizal Fungi on Nitrogen Dynamics During *Cinnamomum camphora* Litter Decomposition

**DOI:** 10.3390/microorganisms13010151

**Published:** 2025-01-13

**Authors:** Yuehong Gao, Xiaoyu Long, Yiqi Liao, Yonghui Lin, Zaihua He, Qin Kong, Xiangshi Kong, Xingbing He

**Affiliations:** 1 College of Biology and Environmental Sciences, Jishou University, Jishou 416000, China; gaoyuehong0720@163.com (Y.G.); 17674363089@163.com (X.L.); linyonghui4849@163.com (Y.L.); hezh@jsu.edu.cn (Z.H.); kongqin0201@163.com (Q.K.); 2 Key Laboratory for Ecotourism of Hunan Province, School of Tourism, Jishou University, Jishou 416000, China; sysylll@outlook.com

**Keywords:** arbuscular mycorrhizal fungi, extracellular enzyme activity, litter decomposition, nitrogen mineralization, protein degradation

## Abstract

Arbuscular mycorrhizal fungi (AMF) can preferentially absorb the released ammonium (NH_4_^+^) over nitrate (NO_3_^−^) during litter decomposition. However, the impact of AMF’s absorption of NH_4_^+^ on litter nitrogen (N) decomposition is still unclear. In this study, we investigated the effects of AMF uptake for NH_4_^+^ on litter N metabolic characteristics by enriching NH_4_^+^ via AMF suppression and nitrification inhibition in a subtropical *Cinnamomum camphora* forest. The results showed that AMF suppression and nitrification inhibition significantly decelerated litter decomposition in the early stage due to the repression of NH_4_^+^ in extracellular enzyme activity. In the late stage, when soil NH_4_^+^ content was low, in contrast, they promoted litter decomposition by increasing the extracellular enzyme activities. Nitrification inhibition mainly promoted the utilization of plant-derived N by promoting the degradation of the amide I, amide II, and III bands by increasing protease activity, and it promoted ammonification by increasing urease activities, whereas it reduced the utilization of microbial-derived N by decreasing chitinase activity. On the contrary, AMF suppression, which significantly reduced the ammonification rate and increased the nitrification rate, only facilitated the degradation of the amide II band. Moreover, it intensified the microbial-derived N decomposition by increasing chitinase activity. The degradation of the amide I and II bands still relied on the priming effects of AMF on soil saprotrophs. This was likely driven by AMF-mediated phosphorus (P) mineralization. Nutrient acquiring, especially P by phosphatase, were the main factors in predicting litter decomposition and protein degradation. Thus, AMF could relieve the end-product repression of locally enriched NH_4_^+^ in extracellular enzyme activity and promote early-stage litter decomposition. However, the promotive effects of AMF on litter protein degradation and NH_4_^+^ release rely on P mineralization. Our results demonstrated that AMF could alleviate the N limitation for net primary production via accelerating litter N decomposition and reducing N loss. Moreover, they could restrict the decomposition of recalcitrant components by competing with saprotrophs for nutrients. Both pathways will contribute to C sequestration in forest ecosystems, which advances our understanding of AMF’s contribution to nutrient cycling and ecosystem processes in subtropical forests.

## 1. Introduction

Decomposition of plant litter is an important biological process for organic carbon (C) release and nutrient recycling [1,2]. The decomposition process is directly influenced by both biological and abiotic factors, such as the climate, litter quality, soil biota, and soil physicochemical properties [2]. Nitrogen (N), as an essential nutrient for the productivity of ecosystems, is frequently of limited availability to plants in most terrestrial ecosystems [3]. Thus, the degradation and recycling of N within plant litter is an crucial step for ecosystem productivity [4]. Macromolecular organic N (e.g., proteins) constitutes the main component of litter N and accounts for more than 60% of nitrogenous substances in litter [5]. Under the action of microorganisms (e.g., bacteria, fungi, and actinomycetes), litter protein is first degraded by protease into peptides, subsequently transformed into ammonium N (NH_4_^+^-N) via ammonification, and further transformed via nitrification into nitrate N (NO_3_^−^-N) [6,7]. The available mineral N is successively taken up and utilized by plants and soil microorganisms [8]. Global climate change (such as warming, N deposition, species invasion, etc.) will lead to the replacement of ectomycorrhizal forests by arbuscular mycorrhizal fungi (AMF) forests, which are dominated by an inorganic nutrient economy for N [9,10]. This will cause changes in important ecological functions, such as forest C sequestration, and it highlights the necessity of incorporating AMF-mediated N mineralization into Earth system models.

AMF can form symbiotic associations with approximately 72% of vascular plants and can develop a comprehensive network of mycorrhizal hyphae for scavenging soil nutrients [11]. In AM-dominated stands, the fast decomposition of high-quality litter pools and elevated rates of C and N mineralization result in an inorganic nutrient economy [9]. As an effective N acquisition strategy, AMF usually colonize decomposing plant litter and quickly take up the newly released N, which is subsequently transferred to the host plants [12,13]. In tropical and subtropical forest ecosystems, most nutrients are taken directly from decomposing litter, and most tropical and subtropical plants form symbiotic associations with AMF [9,14]. Through the mycorrhizal pathway, plants can achieve efficient utilization of N from litter decomposition. AMF lacks saprophytic ability and cannot mineralize organic matter independently [15]; they rely on stimulating saprophytic microorganisms to decompose litter and obtain N [16,17,18]. However, the impact of AMF’s absorption of N on the microbial activity and subsequent ecological processes—such as litter decomposition and N mineralization—in forest ecosystems is still unexplored.

Soil microbes, including bacteria and other fungi, are the primary agents of litter decomposition [2]. Microbial decomposers usually utilize extracellular enzymes to decompose plant residues to obtain N [19]. When bioavailable N is adequate, the extracellular enzyme activity for litter decomposition can be repressed [8]. For example, the addition of NH_4_^+^ leads to a decrease in the activity of peroxidase [20]. Furthermore, activities of extracellular enzymes related to N mineralization are also reduced to varying degrees [21,22]. The addition of NH_4_^+^ reduces protease activity by about 50% [21,23], glycine aminopeptidase activity by about 35% [22], urease activity by up to 95% [24], and amino acid oxidase activity by 30% [25]. This inhibitory effect can be attributed to the end-product repression of NH_4_^+^ [8] or the toxic effects of elevated NH_4_^+^ contents on microorganisms [26] or the tendency of microbial decomposers to preferentially utilize readily available NH_4_^+^ instead of decomposing organic N. The litter decomposition process usually generates excess NH_4_^+^-N in litter microsite, which may affect the activity of microbial decomposers, thereby influencing the litter decomposition and N releasing.

In general, AMF are more competitive at scavenging NH_4_^+^ than other microorganisms, especially ammonia-oxidizing bacteria [27]. The rapid uptake of NH_4_^+^ by AMF hyphae from colonized litter can reduce the NH_4_^+^ concentration in litter microsite. This may lead to the de-repression of NH_4_^+^ in extracellular enzyme activities and promote N release. Previous studies have documented that AMF inoculation can significantly increase the activities of cellulase [28], urease, and protease [29,30], as well as phosphatase [31]. Furthermore, by enriching local NH_4_^+^, the addition of a nitrification inhibitor significantly inhibits the AMF-mediated organic matter decomposition [32]. Therefore, timely removal of newly released NH_4_^+^ from decomposing litter microsite by AMF likely releases saprotrophs from metabolic repression. This may have a positive effect on protein degradation and N release during litter decomposition, but there remains a lack of relevant research on this topic.

In this study, a litter decomposition experiment was conducted in a subtropical evergreen broad-leaved forest of Hunan Province, China’s central region. The leaf litter of *Cinnamomum camphora* L. was subjected to NH_4_^+^ enrichment by suppressing AMF activity with benomyl to block the AMF pathway of NH_4_^+^ transport and/or by decreasing N loss by inhibiting nitrification with dicyandiamide (DCD). Benomyl can effectively suppress the growth and activity of AMF, reducing AMF colonization and spore density [33], but it has little effect on other microorganisms [34]. DCD can effectively inhibit nitrification by blocking the active site of ammonia monooxygenase, thereby significantly increasing soil NH_4_^+^-N content, but it has no significant effect on the general soil microbial community [35,36]. Moreover, DCD has no effect on plant growth and AMF’s colonization of roots and cannot affect the mycorrhizal pathway of N acquisition by plants [27,32]. By tracking the dynamics of litter protein degradation and N mineralization during the decomposition process, this study aimed to investigate the impact of AMF’s absorption of N on the metabolic characteristics of litter N. We hypothesized that (1) nitrification inhibition will decelerate litter decomposition due to end-product repression of extracellular enzyme activity by enriched NH_4_^+^, while AMF’s absorption of N could relieve this effect and promote litter decomposition; and (2) litter protein degradation would be promoted by AMF as they can relieve the end-product repression of locally enriched NH_4_^+^ in extracellular enzyme activity.

## 2. Materials and Methods

### 2.1. Study Site Description and Litter Preparation

The experiment was conducted in the *C. camphora* forest (110°27′28.80″ E, 29°08′37.59″ N) in the back mountain of Zhangjiajie Campus of Jishou University, Hunan Province. This region belongs to the subtropical mountain monsoon humid climate, with an altitude of 250 m, sufficient light and heat, abundant rainfall, a long frost-free period, and a short severe cold period. The annual average sunshine, temperature, and precipitation are about 1440 h, 16 °C, and 1400 mm, respectively. The average frost-free period is between 216 days and 269 days, and the annual average relative humidity of the forest is 70% [37]. The forest is dominated by *C. camphora*, with abundant surface shrubs and herbaceous plants. The soil is a slightly acidic humic, with a water content of 21.26%, a NO_3_^−^ content of 10.21 mg kg^−1^, a NH_4_^+^ content of 14.69 mg kg^−1^, an available phosphorus (P) content of 23.72 mg kg^−1^, and a pH of 5.42 ± 0.05 [37].

Nowadays, natural forests have been severely damaged by human activities, and artificial forests are the main means of forest ecosystem restoration. *Cinnamomum camphora* L., as a representative tree species of artificial forests, is widely distributed in the subtropical region of China and plays an important role in regional soil and water conservation, as well as in the C cycle. The spring equinox is its main defoliation period. Before the start of the experiment, a collection net was set up in the *C. camphora* forest, and the fallen leaves of the *C. camphora* were collected during the defoliation period in March. The leaf litter was taken back to the laboratory, dried to constant weight at 50 °C, and packed into 20 cm × 20 cm nylon litterbags at the standard of 7 g of dried leaves per bag. The mesh size of the litterbags was 1 mm, which could exclude soil macrofauna that have a large range of motion and strong grazing effects [38].

### 2.2. Experimental Design

This study conducted a full factorial experiment of AMF suppression and nitrification inhibition. The litter was assigned into 4 groups: (1) deionized water was sprayed as control (CK); (2) DCD was applied to inhibit nitrification (DCD) while allowing the AMF pathway to transport NH_4_^+^-N; (3) benomyl was applied to suppress AMF activity (B); (4) benomyl and DCD were applied simultaneously (B + DCD). In a 20 × 20 m *C. camphora* forest plot, five 5 × 5 m subplots (5 replicates) were established in March 2023. Within each subplot, four 1 × 1 m blocks were set up with a spacing of 1–2 m between adjacent blocks to ensure that there was no interference between the blocks. Litterbags were randomly tiled in the blocks, with 20 per block, for a total of 400 litterbags. During the experiment, 1.25 L (1.2 g L^−1^ effective content) of benomyl and dicyandiamide were sprayed every 40 days for AMF suppression and/or nitrification inhibition groups, and the same volume of deionized water was sprayed in the control [37]. In total, 100 litter samples were retrieved every three months during the one-year decomposition period, and 5 litterbags in each block for each treatment were collected. Soil samples under the litterbags were collected, sifted through a 2 mm sieve, and stored in a 4 °C freezer for analysis.

### 2.3. Measurement of Mass Loss, Mycorrhiazl Colonization, and Protein Degradation

The remaining litter mass was determined using the oven-drying method and calculated as a percentage of the initial mass. Mass loss was calculated as the difference between the two sampling times. The roots that beneath the litterbags were collected, washed free of soil, cut into 1 cm, cleared with 10% KOH in a 90 °C water bath, and stained in acid fuchsin. The rate of mycorrhizal colonization was calculated using the gridline intersect method [37]. The decomposing litter fragments were also checked for AMF colonization. Stained aseptate hyphae with characteristic unilateral angular projections were considered mycorrhizal.

Protein degradation was assessed using Fourier transform infrared spectroscopy (FTIR, Bruker Corporation, Berlin, Germany), according to Li et al. [39]. The decomposing litter was ground into a powder and sifted through a 200-mesh sieve. For the preparation of potassium bromide (KBr) pellets, the litter powder was mixed with KBr at a ratio of 1:50. The prepared KBr pellets were then scanned 16 times in the range of 400 to 4000 cm^−1^ utilizing a Fourier transform infrared spectrometer, with a resolution of 4.0 cm^−1^. The infrared spectrum was preprocessed, and the baseline was corrected using Omnic 9.2 (Thermo Fisher Scientific, Massachusetts, USA) and then plotted in Origin 2022 (OriginLab, Massachusetts, America). The absorption peaks within the range of 1700 to 1610 cm^−1^ correspond to the amide I band; those within the range of 1570 to 1510 cm^−1^ correspond to the amide II band; and the amide III band is found within the range of 1335 to 1200 cm^−1^ [40,41]. These amide bands represent the absorption peaks associated with protein peptide bonds. The peak areas of the amide bands were divided by the sum areas of all absorption peaks obtained to give the relative peak area [42]. Changes in the relative peak areas of these bands indicated the protein degradation dynamic.

### 2.4. Measurement of Extracellular Enzyme Activities and N Mineralization

Enzyme activities involved in C cycling (cellulase or carboxymethyl cellulase (Cx), invertase (Inv), and β-1,4-glucosidase (BG)), protein degradation (protease (Pro) and chitinase or β-N-acetylglucosaminidase (NAG)), ammonification (urease, Ure), and P cycling (acid phosphatase (ACP) and alkaline phosphatase (AKP)) in soil were determined spectrophotometrically with little modification (see Table S1 for the detailed assay procedure). Soil samples were stored under 4 °C, and enzymatic activities were assayed within one week of sampling.

Soil water content (Swc) was determined using the oven-drying method. Soil pH was measured with a pHS-25 pH meter (Leici, Shanghai, China) at a water-to-soil ratio of 2.5:1. Soil N mineralization was determined according to Trap et al. [43]. After passing through a 2 mm sieve, the fresh soil samples equivalent to 10 g of dry soil were weighed and put into triangular flasks. The contents of NO_3_^−^-N and NH_4_^+^-N were determined via phenol disulfonic acid and indigo phenol blue spectrophotometry, respectively [44].

### 2.5. Statistical Analyses

The Olson exponential decay model was used to estimate the litter decomposition rate [45], i.e., *M_t_*/*M*_0_ = *e*^−*kt*^, where *M_t_* is the litter remaining mass after *t* months of decomposition; *M*_0_ is the initial litter mass; *t* is the decomposition time (month); and *k* is the litter decomposition coefficient. The net ammonification (*R_amm_*), nitrification (*R_nit_*), and mineralization (*R_min_*) rates were calculated according to Trap et al. [43]: *R_amm_* = (NH_4_^+^-N_t_ − NH_4_^+^-N_0_)/*t*; *R_nit_* = (NO_3_^−^-N_t_ − NO_3_^−^-N_0_)/*t*; and *R_min_* = [(NH_4_^+^-N_t_ + NO_3_^−^-N_t_) − (NH_4_^+^-N_0_ + NO_3_^−^-N_0_)]/*t*, where NH_4_^+^-N_t_ and NO_3_^−^-N_t_ represent the amounts of NH_4_^+^-N and NO_3_^−^-N after 7 days of incubation (post-incubation), respectively; NH_4_^+^-N_0_ and NO_3_^−^-N_0_ represent their initial contents before incubation (pre-incubation); and *t* is the incubation time (day). The results are expressed in mg·kg^−1^·d^−1^. The effect size of nitrification and/or AMF suppression were calculated by dividing the measured variables under suppression treatments by the measured variables under control, where an effect size greater than 1.0 indicates a positive effect, while an effect size less than 1.0 indicates no effect or a negative effect.

The data were analyzed for variance using the “EasyStat” package in R (4.0.5). The multiple comparison was used to analyze the differences between the two pairs through post hoc Tukey’s honest significant difference (HSD) test at *p* < 0.05. Repeated measures ANOVA was used to compare the impacts of nitrification inhibition and AMF suppression on extracellular enzyme activity and soil physicochemical properties. Principal component analysis (PCA) using the “vegan” package in R and the heatmap of Spearman correlation were conducted to reveal the relationships. Random forest analysis was used to predict the importance of factors affecting litter mass loss and protein degradation using “randomForest” package in R.

## 3. Results

### 3.1. Decomposition Dynamics of C. camphora Litter

**Figure 1:**
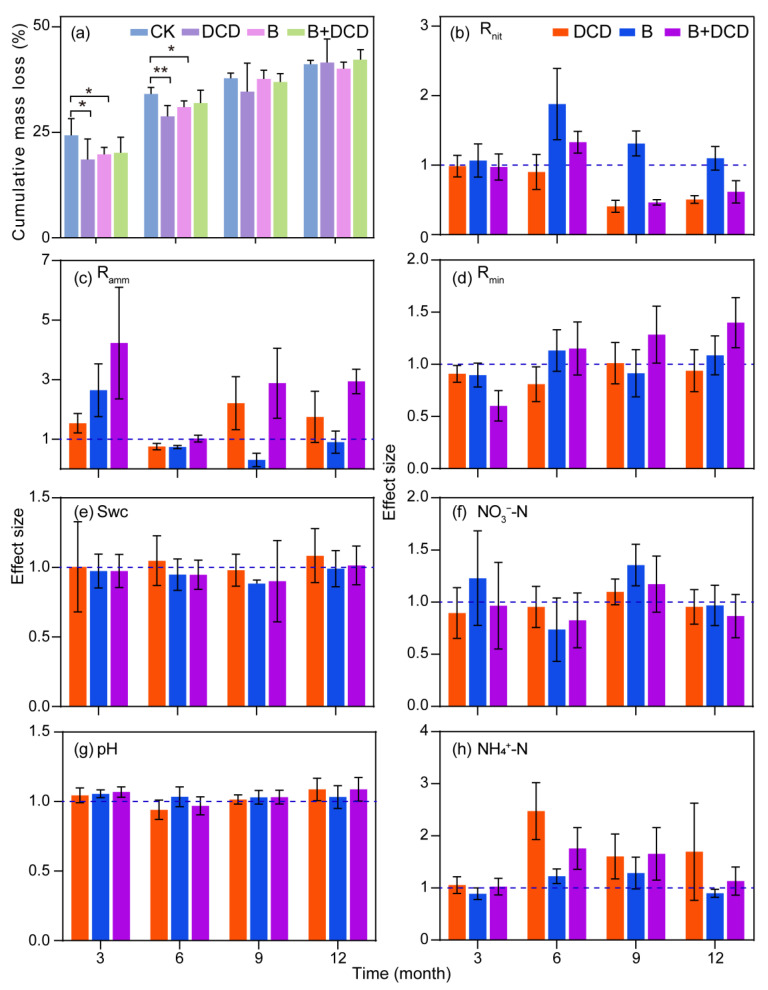
Dynamics of mass loss and soil physicochemical properties during one year of litter decomposition under different conditions. CK: deionized water was sprayed as control; DCD: dicyandiamide was applied to inhibit nitrification; B: benomyl was applied to suppress AMF activity; B + DCD: benomyl and dicyandiamide was applied simultaneously; Swc: soil water content. Values are the means and standard deviation for (**a**), and 95% confidence intervals for (**b**–**h**) (n = 5). * and ** in (**a**) indicate differences at the 0.05 and 0.01 levels, respectively, and intervals in (**b**–**h**) that do not cross the 1.0 dashed line indicate significant effects.

After one-year of decomposition, the cumulative mass loss of *C. camphora* litter ranged from 40% to 42% (Figure 1a). The root colonization ranged from 38% to 74% (Figure S1). Benomyl application significantly decreased the mycorrhizal colonization percentage, whereas DCD application significantly increased the mycorrhizal colonization percentage (Tukey test, *p* < 0.05). During the first six months, *C. camphora* litter decomposed rapidly, accompanied by a notable decline in the mass loss over time. The cumulative mass loss in AMF suppression and nitrification inhibition treatments was significantly lower than that of the control (Tukey test, *p* < 0.05) (Figure 1a). During the last six months, *C. camphora* litter decomposed slowly, and at the end of the experiment, the difference with control disappeared (Figure S2). The fitting results of the Olason exponential model showed that it took 12.65 and 54.69 months for 50% and 95% of the litter to be decomposed under natural conditions, respectively (for details, see Supplementary Table S2). After AMF was suppressed, the time was extended by 6.80% and 6.75%, respectively. After nitrification was inhibited, the time increased by 11.30% and 11.25%, respectively. When both nitrification and AMF were suppressed, the time was increased by 4.58% and 4.52% compared to that of the control, respectively.

### 3.2. Soil Nitrogen Mineralization Dynamic

Repeated measures ANOVA showed that nitrification inhibition, AMF suppression, and their interactions significantly affected the soil N mineralization process (*F*-test, *p* < 0.05) (Table 1). During the initial three months, the net ammonification rate was negative, while the net nitrification rate was the highest over the whole decomposition period (for details, see Figure S2). After AMF was suppressed, the net mineralization rate during the initial three months and the net ammonification rate over the entire period decreased significantly, but the net nitrification rate increased significantly (Tukey test, *p* < 0.05) (Figure 1b–d, and Figure S2). After nitrification was inhibited, the ammonification in the final six months was enhanced significantly (Tukey test, *p* < 0.05) (Figure 1c). Moreover, the net nitrification rate in the nitrification inhibition groups was reduced significantly comparing to the non-inhibition groups in the final six months (Tukey test, *p* < 0.05). The interaction of nitrification inhibition and AMF suppression showed a synergistic effect on the mineralization and ammonification rates (*F*-test, *p* < 0.05) (Table 1). Simultaneously inhibited AMF and nitrification significantly increased the net mineralization and ammonification rates after three months of decomposition (Tukey test, *p* < 0.05) (Figure 1c,d).

Soil water content, NO_3_^−^-N content, and pH all increased during the first 6 months of decomposition (for details, see Figure S2). The soil NH_4_^+^-N content was the highest during the initial three months and decreased rapidly afterward. The contents of NO_3_^−^-N, NH_4_^+^-N, and pH all showed seasonal changes with respect to water content, indicating that soil water content affected the changes of soil pH and nutrient contents. The suppression of AMF had a statistically significant impact on soil pH (*F*-test, *p* < 0.05); specifically, both nitrification inhibition and AMF suppression resulted in an increase in soil pH (Figure 1g). Repeated measures ANOVA showed that nitrification inhibition significantly affected soil NH_4_^+^-N (*F*-test, *p* < 0.05) (Table 1), while the interaction of nitrification inhibition and AMF suppression showed an antagonistic effect on NH_4_^+^-N content (*F*-test, *p* < 0.05). Throughout the entire decomposition period, nitrification inhibition markedly increased the soil NH_4_^+^-N content and obviously reduced NO_3_^−^-N content (Tukey test, *p* < 0.05, Figure 1f,h). However, significant effects of AMF suppression on NO_3_^−^-N and NH_4_^+^-N depended on decomposition time (*F*-test, *p* < 0.05).

### 3.3. Dynamics in Extracellular Enzyme Activity

**Figure 2:**
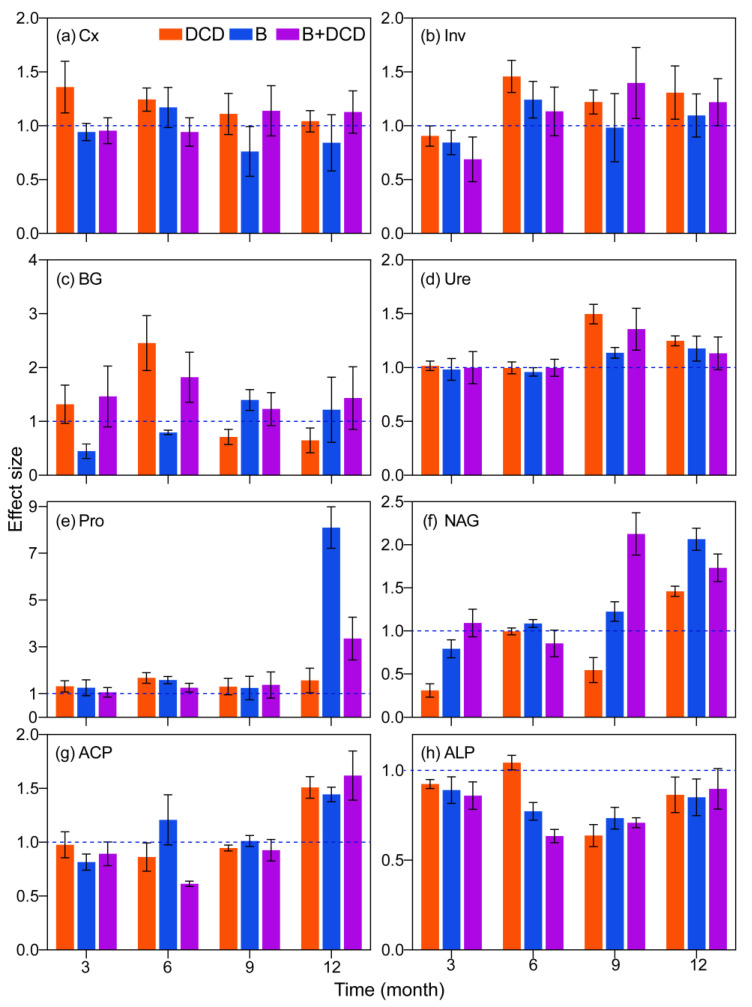
Effects of nitrification inhibition and AMF suppression on extracellular enzyme activity during one year of litter decomposition. Values are means and 95% confidence intervals (n = 5). Intervals that do not cross the 1 dashed line indicate significant effects. (**a**–**h**) indicate the activities of carboxymethyl cellulase (Cx), invertase (Inv), β-1,4-glucosidase (BG), urease (Ure), protease (Pro), β-N-acetylglucosaminidase (NAG), acid phosphatase (ACP), and alkaline phosphatase (ALP), respectively.

During the whole decomposition period, the extracellular enzyme activities differed significantly in different stages (*F*-test, *p* < 0.05) (Table 1). Except for Ure, most enzyme activities were obviously lower in the initial three months than later months (for details, see Figure S3). Nitrification inhibition significantly increased the Cx activity over the whole period and Inv activity after three months (Tukey test, *p* < 0.05) (Figure 2a,b). However, it increased BG activity during the first six months and decreased it afterward (Tukey test, *p* < 0.05) (Figure 2c). AMF suppression decreased Cx activity during the whole period and BG activity during the early six months (Tukey test, *p* < 0.05) (Figure 2a,c). Nitrification and/or AMF suppression significantly increased Ure activity in the late six months and Pro activity over the whole period (Tukey test, *p* < 0.05) (Figure 2d,e). AMF suppression increased NAG activity, while nitrification inhibition decreased it (Tukey test, *p* < 0.05) (Figure 2f). The interaction of nitrification inhibition and AMF suppression showed an antagonistic effect on ACP activity (*F*-test, *p* < 0.05). Nitrification and/or AMF suppression significantly decreased phosphatase activity, while only increasing ACP activity at the end of the period (Tukey test, *p* < 0.05) (Figure 2g,h). ALP activity decreased obviously over time (Tukey test, *p* < 0.05) (Figure S3). Except for phosphatase, simultaneous inhibition of nitrification and AMF significantly increased the activities of most extracellular enzymes after three months, especially BG activity (Tukey test, *p* < 0.05) (Figure 2 and Figure S3).

### 3.4. Dynamics in Litter Protein Degradation

**Figure 3:**
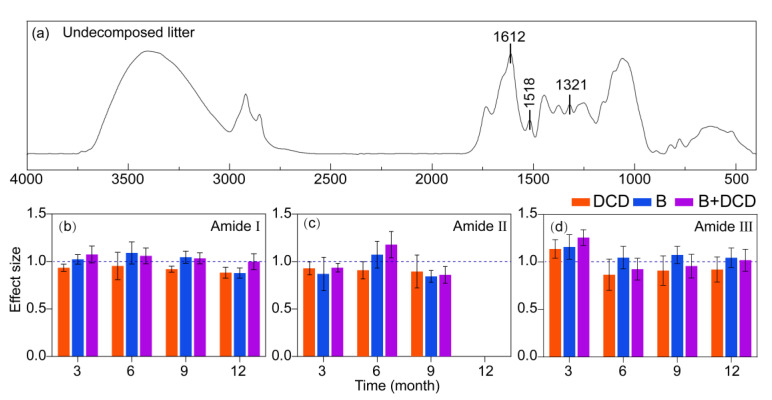
Infrared spectral characteristics of litter protein degradation under different conditions. (**a**) indicates the infrared spectral characteristics of the undecomposed litter, and (**b**) to (**d**) indicate the effects of treatments on the relative peak area of the amide I (**b**), II (**c**), and III (**d**) bands, respectively. Values of effect size from (**b**) to (**d**) are mean and 95% confidence intervals (n = 5). Intervals that do not cross the 1.0 dashed line indicate significant effects.

At various stages of decomposition, the infrared spectra of *C. camphora* litter, including the undecomposed litter, exhibited nine distinct peak ranges (Figure 3a and Figure S4). After three months of decomposition, a peak indicative of an irregular coiled structure (1642–1649 cm^−^¹) [46] was observed within the range of the amide I band in the AMF suppression treatment (Figure 3a and Figure S4), whereas this structure emerged after six months of decomposition in the control and nitrification inhibition treatments. The irregular curled structure was no longer present in the nitrification inhibition treatment by the ninth month. In contrast, the irregular curled structure persisted in the other treatments (Figure S4).

After twelve months of decomposition, the irregular curled structures of all the treatments disappeared or were not obvious, and the peaks in the amide II band of all treatments disappeared (Figure 3c and Figure S4). Following the inhibition of nitrification, the relative peak area of the amide I, amide II, and amide III bands was observed to be lower compared to control (Tukey test, *p* < 0.05) (Figure 3). After three months, the relative peak area within amide II for both THE control and nitrification inhibition treatments was greater than that of AMF suppression treatment (Tukey test, *p* < 0.05) (Figure 3c and Figure S4). in particular, after nine months of decomposition, the relative peak area associated with the AMF suppression was the lowest (Figure 3b). However, the relative peak area within the amide I and III bands for the AMF suppression groups was found to be greater than that of the non-AMF suppression groups along the entire decomposition period (Tukey test, *p* < 0.05) (Figure 3b, d).

### 3.5. The Relationships Among Litter and Soil Physicochemical Properties

**Figure 4:**
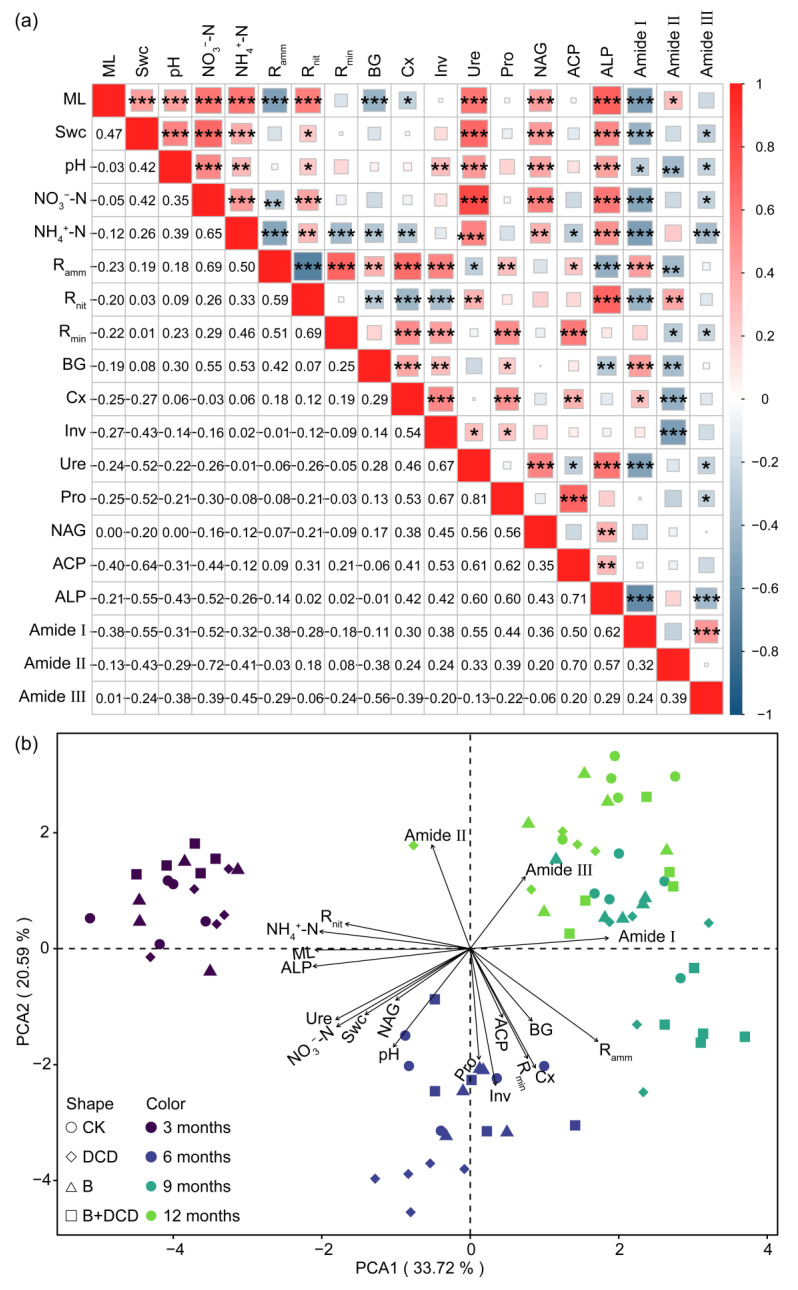
Correlation heatmap (**a**) and principal component analysis (**b**) of mass loss, soil physicochemical properties, and microbial activity. The values in the lower left of (**a**) are the Spearman correlation, and *, **, and *** in the upper right indicate significant difference at the 0.05, 0.01, and 0.001 levels, respectively. The abbreviations are the same as those in Table 1.

Spearman correlation showed that mass loss was positively correlated with Swc, pH, inorganic N contents, and N and P cycling-related enzyme activities, while it was negatively correlated with liable C cycling enzyme activities (*t*-test, *p* < 0.05) (Figure 4a). Swc was positively correlated with pH, inorganic N contents, *R_nit_*, and the activities of Ure, NAG, and ALP (*t*-test, *p* < 0.05). *R_amm_* was positively correlated with BG, Cx, Inv, Pro, and ACP activities, while it was negatively correlated with Ure and ALP activities (*t*-test, *p* < 0.05). *R_nit_* was positively correlated with Ure and ALP activities, while it was negatively correlated with BG, Inv, and Cx activities (*t*-test, *p* < 0.05). *R_min_* was positively correlated with the activities of most enzymes and *R_amm_* (*t*-test, *p* < 0.05). Moreover, inorganic N contents were positively correlated with N and P cycling-related enzyme activities, while they were negatively correlated with liable C cycling enzyme activities (*t*-test, *p* < 0.05). The relative peak area of amide I, II, and III showed negative correlation with most of the soil physicochemical properties and extracellular enzyme activities (*t*-test, *p* < 0.05).

**Figure 5:**
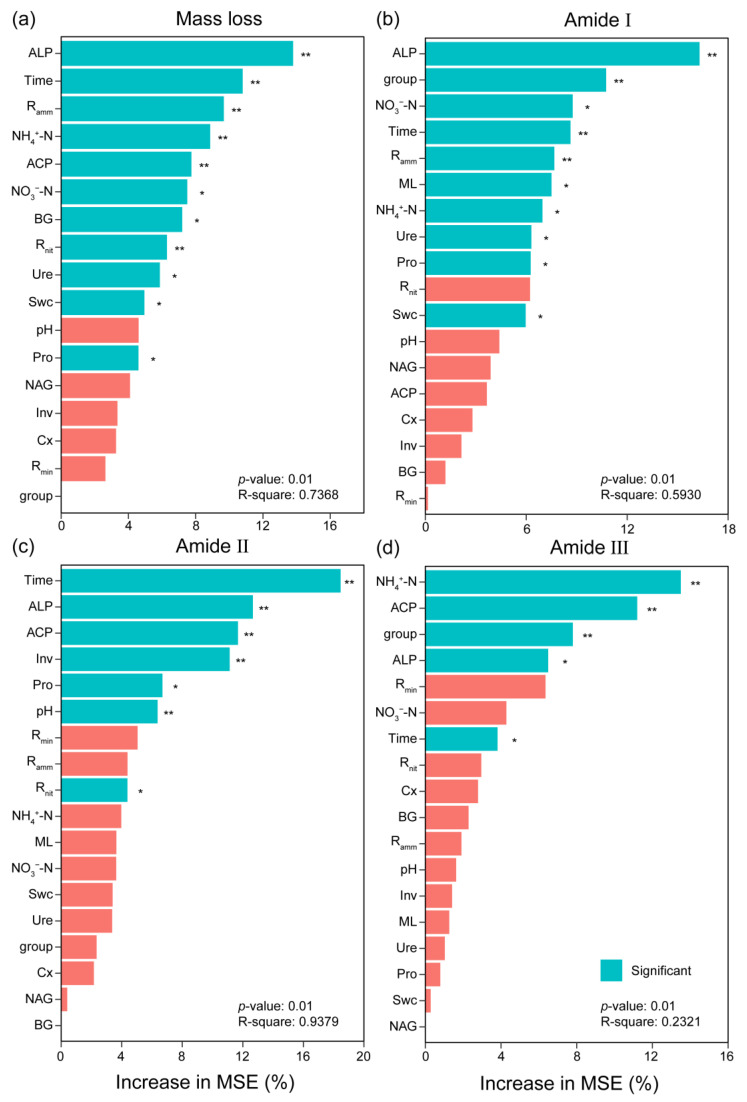
Random forest analysis for identifying the relative contribution of soil physicochemical properties and extracellular enzyme activity to the variation of mass loss (**a**) and the relative peak area of the amide I (**b**), amide II (**c**), and amide III (**d**) bands by their importance value. Predictor importance was estimated by the Gini index, with higher values indicating greater significance. * and ** indicate difference at 0.05 and 0.01 levels via Kruskal–Wallis’s test, respectively. The abbreviations are the same as those in Table 1.

In principal component analysis (PCA), the first two axes explained 33.72% and 20.59% of the variation (Figure 4b). The first axis mainly isolated the early decomposition stage (the early six months) from the late decomposition stage (from September to March of the next year). Mass loss, ALP, NH_4_^+^-N, *R_nit_*, and amide I were highly correlated with PC1. Pro and Inv activities were highly correlated with PC2. Swc was positively correlated with Ure, NAG, NO_3_-N, and pH value. BG and Cx was positively correlated with *R_min_* and *R_amm_*. PCA ordination characterized and distinctly separated the decomposition stages. Different relationships occurred in different decomposition stages (Figures S5 and S6). Random forest regression showed that phosphatase activities, especially ALP, were the primary factor for predicting mass loss and protein degradation (Figure 5). The importance of other predictors for mass loss included time, *R_amm_*, NH_4_^+^-N, ACP activity, NO_3_^−^-N, BG activity, *R_nit_*, Ure activity, Swc, and Pro activity, which decreased in the following order. Other important predictors for protein degradation contained activities of Ure, Pro, Inv, and Cx, as well as NO_3_-N and NH_4_^+^-N (Figure 5b–d).

## 4. Discussion

### 4.1. Litter Decomposition

Lacking a saprophytic ability, AMF mainly promote litter decomposition by stimulating soil saprotrophs to decompose plant litter and obtain the released N, the process of which depends on soil nutrient conditions [28,30,32]. Consistent with these studies, the priming effects of AMF on soil saprotrophs was blocked after AMF suppression, displayed via the reduction in the activities of β-glucosidase, carboxymethyl cellulase, and invertase. Thus, the decomposition of *C. camphora* litter was slowed down, as indicated by the extended half-life and total decay period. However, the significant deceleration of litter decomposition was observed only in the early stage. The timely removal of NH_4_^+^-N by AMF might be another means of promoting litter decomposition. Cheng et al. [32] showed that enriching local NH_4_^+^ significantly offset the promotive impact of eCO_2_ on AMF-mediated organic matter decomposition. In our study, soil NH_4_^+^-N content was high in this stage. When AMF activity was suppressed, their capacity to absorb and transport NH_4_^+^-N was weakened. This resulted in the enrichment of NH_4_^+^-N in the microsites of the decomposing litter, which produced end-product repression of extracellular enzyme activity. The low activities of most of the extracellular enzymes could prove this repression effect. This was also evidenced by the reduction in litter mass loss after nitrification was inhibited. DCD can inhibit the activity of nitrifying bacteria and archaea [47] and reduce N loss via leaching or gaseous N_2_O emissions, thereby enriching soil NH_4_^+^-N content [48,49]. During one year of decomposition, the nitrification inhibition groups had higher soil NH_4_^+^-N compared to other treatments. This led to the limited activities of extracellular enzymes and, subsequently, decelerated litter decomposition. This was consistent with our first hypothesis. High NH_4_^+^ content was reported to significantly reduced the relative abundance and richness of saprophytic fungal community [50,51]. Thus, alongside the priming effect, AMF’s absorption of N from decomposing litter can also promote litter decomposition by releasing saprotrophs from metabolic repression. However, this effect was weakened after nitrification was inhibited, as we found that the DCD treatment had the lowest mass loss. Both the short- and long-term (7-year) application of DCD showed that DCD had little effect on the diversity of soil bacterial communities and did not affect other non-target microbial and enzyme activities [36,52]. Thus, DCD may inhibit the N cycling microbes in the AMF hyphosphere microbiome [53], and it blocks its promotion effects on litter decomposition, which needs further research.

However, in the late stage, the differences in cumulative mass loss between nitrification inhibition and AMF suppression with control were shortened, and their negative effects on litter decomposition in the early stage were offset. This suggested that nitrification inhibition and AMF suppression, on the contrary, promoted the decomposition of *C. camphora* litter in the late stage. This was partially inconsistent with our first hypothesis. With litter decomposition progressing, soil NH_4_^+^-N was continuously depleted, and its content decreased, resulting in the removal of its repression of extracellular enzyme activity. What is more, this low NH_4_^+^-N content had become a factor restricting the growth and activity of saprotrophs, thereby limiting their capacity in litter decomposition. Thus, there is a positive correlation between mass loss and inorganic N contents, as well as N and especially P cycling-related enzyme activities, while there is a negative correlation with liable C cycling enzyme activities. This indicates that nutrient acquiring was the main driver in litter decomposition. In this stage, the decomposition of the remaining recalcitrant components (e.g., lignin) requires more energy and nutrients. AMF, in turn, competes with soil microbial communities for nutrients and inhibits the development of soil fungal and bacterial groups [17]. Suppression of AMF activity could weaken this competition, and it promoted the accumulation of NH_4_^+^-N in soil [32] such that the soil saprotrophs could use these N sources to grow and synthesize extracellular enzymes, thereby promoting litter decomposition. Therefore, AMF suppression and nitrification inhibition in the late stage was observed to stimulate litter decomposition. Our results were consistent with those of previous studies that indicated that AMF had no or negative effects on recalcitrant litter decomposition [54,55].

However, the rate of litter decomposition is influenced by a variety of factors [56]. As an essential transport medium for substrates, soil water content affects the activity of microbial decomposers greatly [37,57]. Excess soil water content results in limited O_2_ diffusion, which will reduce the activity of aerobic microorganisms, but which could increase the activities of anaerobes [58]. Low soil moisture decreases microbial activity by reducing the diffusion of soluble substrates, microbial mobility, and intracellular water potential [58]. In our study, soil water content (below 32%) (Figure S1) showed a positive relationship with mass loss, most of the soil physicochemical properties, and nutrient-acquiring enzyme activities. Relatively higher soil water content could promote the leaching process of labile C and nutrients, which support microbial growth, especially in early-stage decomposition [37]. Moreover, relatively higher soil water content facilitates the transport of low-mobility NH_4_^+^ and alleviates the heterogeneity of NH_4_^+^ distribution, which mitigates nutritional imbalance for saprotrophs, thereby promoting litter decomposition [59,60]. It is reported that AMF can act as a soil conditioner for improving soil water retention and hydraulic conductivity by enhancing soil aggregation [61]. The regulation of AMF with respect to soil water potential encourages drainage in loams and enhances water storage in sands [61], which may also facilitate litter decomposition. But this needs further investigation.

### 4.2. Soil N Mineralization Dynamics

The release of litter nutrients is affected by combinations of soil nutrient availability, litter quality, and environmental conditions during the decomposition process [62]. Over one year of decomposition, except for NH_4_^+^-N, soil NO_3_^−^-N content and pH showed seasonal variation with water content. In the first six months, the decomposing litter experienced a waring summer, and the N transformation by microorganisms shifts from assimilation to dissimilation, thereby promoting the nutrient release [63]. Relatively high soil NH_4_^+^-N content in the early stage might be due to the fast decomposition of *C. camphora* litter [37]. During this stage, microorganisms might engage in N immobilization, that is, using NH_4_^+^-N to synthesize organic N for reproduction and growth [64]. This led to the negative net ammonification rate in the first three months of litter decomposition. AMF extraradical hyphae show a preference for NH_4_^+^-N, and their absorption and transfer capacity for NH_4_^+^-N is generally higher than that of NO_3_^−^-N [65]. This led to the relatively lower NH_4_^+^-N content in control than other treatments (Figure 1), which directly reduced NH_4_^+^ leaching and volatilization. Lower soil NH_4_^+^ concentrations also suppress nitrification, resulting in reduced NO_3_^−^ leaching losses [66].

In our study, we found that after AMF was suppressed, the ammonification was inhibited over the entire period, and the net nitrification rate was increased significantly. This indicated that AMF, on the one hand, increases ammonification and promotes litter N release, and on the other hand, it prevents N loss by suppressing nitrification. AMF can stimulate the activity of ammonification microorganisms through priming effects and compete with nitrifying bacteria for NH_4_^+^-N and inhibit nitrification [67,68,69]. Moreover, when soil NH_4_^+^-N content was low during the late decomposition stage, nitrification inhibition also significantly increased the net ammonification rate and decreased the net nitrification rate, which can effectively reduce N loss [48]. Both AMF suppression and nitrification inhibition could increase the N supply for microbial growth [70]. We observed that nitrification inhibition significantly increased the AMF colonization. This, in turn, promoted the conversion of litter organic N to inorganic N. Thus, when both AMF activity and nitrification were inhibited; the net mineralization and ammonification rates were significantly higher than in other treatments after six months of decomposition. There is growing evidence that N availability is declining in many non-agricultural ecosystems such as forests and grasslands worldwide [3]. The rising atmospheric CO_2_ concentration will intensify this progressive N limitation, which constrains the net primary production of the terrestrial ecosystems [71]. AMF could relieve this limitation by reducing N loss through N_2_O and increasing N use efficiency [49,72]. This will promote C sequestration in forest ecosystems.

### 4.3. Extracellular Enzyme Activities

In forest ecosystem, microorganisms decompose organic matter by secreting extracellular enzymes, and the activities of these enzymes are an important index reflecting the changes in microenvironments [73]. During the early stage of decomposition, except for urease, the activities of most extracellular enzymes were relatively low. The reason might be that the relatively high soil NH_4_^+^-N content has end-repression effects on extracellular enzyme activities [20]. AMF effectively absorbs and transport local NH_4_^+^-N and increases β-glucosidase activity, thus accelerating litter decomposition, which is consistent with previous studies [30,74,75].

After three months of decomposition, the soil N NH_4_^+^-N content decreased significantly and, on the contrary, become a factor restricting the activity of microbial decomposers [76]. Inorganic N contents were positively correlated with N and P cycling-related enzyme activities, while they were negatively correlated with liable C cycling enzyme activities. This suggested that microbial decomposers invested more energy and C to release nutrients from decomposing litter. Nitrification inhibition can improve the soil NH_4_^+^-N level by reducing N loss, while AMF suppression can relieve the competition between AMF and microbial decomposers for NH_4_^+^-N [77]. Both pathways contributed to the growth and metabolism of microorganisms, thereby increasing microbial activities [48]. Thus, an increase in β-glucosidase activity in the late decomposition stage and in protease activity throughout the decomposition period was observed after AMF suppression. The activities of protease, cellulase, urease, and invertase were also increased after nitrification inhibition [78]. Furthermore, both AMF suppression and nitrification inhibition significantly decreased phosphatase activity, especially ALP, which was the most important factor for predicting mass loss and protein degradation. Extraradical hyphae of AMF can carry and recruit a specific hyphosphere microbiome to the organic P region, enhancing the mineralization of organic P [79,80]. Suppressing AMF should, unavoidably, decrease phosphatase activity. However, a negative correlation was only observed between NH_4_^+^-N and ACP activity. Thus, alongside end-product repression, other mechanisms may exist regarding the negative effects of nitrification inhibition on phosphatase activity, which is a subject in need of further investigation.

### 4.4. Litter Protein Degradation

In infrared spectroscopy, amide bands are used as characteristic absorption peaks of the amide groups (-CONH-) in proteins and peptides [81]. During the first three months of litter decomposition, there were peaks in the amide I band representing the irregular coiled structure of the proteins. The proteins had been transformed from an ordered structure to a disordered and unstable structure, which could more easily be decomposed by microorganisms [46]. The irregular coil structure disappeared in advance after nitrification was inhibited, indicating that nitrification inhibition could accelerate the degradation of litter proteins. Most extracellular enzyme activities were negatively correlated with the relative peak area of the amide bands, suggesting that higher microbial activity facilitated the degradation of litter protein.

In our study, different mechanisms were observed with respect to nitrification inhibition and AMF suppression in promoting litter protein degradation. Nitrification inhibition increased the activities of protease and urease and promoted the degradation of litter protein, as displayed by the dramatic decrease in the relative peak areas of the amide I, II, and III bands. However, chitinase activity was reduced. Chitinase is capable of breaking down the cell walls of insects and fungi, which is the main source of mineralizable N in soils [82]. These results imply that by enriching soil NH_4_^+^-N content, nitrification inhibition mainly promoted the utilization of plant-derived N by enhancing protein degradation and ammonification while reducing the utilization of microbial-derived N. AMF suppression also increased the activities of protease and urease, thereby promoting protein degradation and ammonification. However, this only facilitated the decomposition of the amide II band. AMF suppression increased soil NH_4_^+^-N levels mainly by reducing NH_4_^+^-N uptake. This could dramatically reduce the competition for N with soil microorganisms in the later stage, i.e., when the soil NH_4_^+^-N content was low. Simultaneously, the labile C subsidy from AMF should also be reduced significantly. These hyphal exudates play important roles in recruiting the hyphosphere microbiome and support the migration of functional specific microorganisms [80,83]. Thus, the stimulatory effects of AMF on microbial decomposers, such as saprophytic fungi with a great ability to decompose lignin, are weakened [28]. This is especially important at the late decomposition stage, i.e., when litter protein is degraded and N is released, mainly via the co-metabolism pathway [84]. This leads to the reduced degradation of litter protein, such as in the amide I and III bands. Moreover, chitinase activity was also increased after AMF was suppressed, suggesting thar the competition among soil microorganisms intensified, likely for liable C [85]. This implies that AMF facilitate the degradation of the amide I and III bands via priming effects, which is inconsistent with our second hypothesis. Phosphatase activities, especially ALP, were negatively correlated with the relative peak areas of the amide I and III bands. This suggests that the promotion effects of AMF on the degradation of the amide I and III bands rely on the P-mineralization microbiome, which is carried or recruited by AMF extraradical hyphae [79,80].

### 4.5. Limitiations of the Study

As a broad-spectrum fungicide, benomyl and its primary degradation product, carbendazim (methyl 2-benzimidazole carbamate), may affect other non-target organisms. Previous studies have shown that benomyl is a risk to soil invertebrates, which may influence nutrient absorption and plant growth [86]. The application of the fungicide fundazol (benomyl’s active ingredient) decreased the relative abundance of actinomycetes and the general group of bacteria and, paradoxically, increased the population of fungi [87]. However, studies have also reported that fundazol did not significantly affect the structure of the soil microbial community. Moreover, benomyl contains N (6%), and its half-life ranges from <2 up to 7 weeks [88]. After decomposition, benomyl may become a source of N that can be used by soil microorganisms [89]. On the other hand, DCD is water-soluble and susceptible to biodegradation via guanylic urea, guanidine, and urea, yielding NH_4_^+^ [90]. This process occurs within a few weeks of application and is intensified under high temperatures [91]. By providing N, DCD is reported to increase the abundance of ammonia-oxidizing archaea in the absence of AMF [27]. Thus, both benomyl and DCD could be served as the potential N sources and interfere with the results.

## 5. Conclusions

Fast decomposition during the early decomposition stage resulted in high soil NH_4_^+^-N content, which decreased extracellular enzyme activity through end-product repression. Nitrification and/or AMF suppression further enriched NH_4_^+^-N content in litter microsites and decelerated the decomposition of *C. camphora* litter. During the late decomposition stage, when the soil NH_4_^+^-N content was low, nitrification inhibition and AMF suppression, in contrast, promoted litter decomposition by enriching NH_4_^+^-N, which provided N for saprotroph growth. Releasing nutrients, especially P by phosphatase, was the main factor in predicting litter decomposition and protein degradation. Nitrification inhibition mainly promoted the utilization of plant-derived N and reduced the utilization of microbial-derived N. On the contrary, by enriching soil NH_4_^+^-N content, AMF suppression only facilitated the degradation of the amide II band. The degradation of the amide I and III bands still relayed on the priming effects of AMF on soil saprotrophs, especially in the late decomposition stage. This was likely driven by AMF-mediated P mineralization. Thus, litter P metabolism should be taken into consideration regarding AMF-mediated litter N mineralization. With the increase in atmospheric CO_2_ concentration, net primary production in forest ecosystems will face the progressive N limitation. Our results demonstrated that AMF could alleviate this N limitation by improving N use efficiency, reducing N loss, and accelerating N mineralization by promoting labile C decomposition. On the other hand, AMF will restrain the decomposition of recalcitrant components by competing with saprotrophs. Both pathways will contribute to C sequestration. Given the intricate roles of AMF and their interactions within the microbial network, it is imperative to conduct further research on the relationships between AMF and other soil microbial decomposers, as well as on the implications of these interactions for the N cycle within the forest ecosystems.

## Figures and Tables

**Table 1:** Repeated measures ANOVA (sampling time as the repeated factor, T) for the effects (F-values) of nitrification inhibition (DCD), AMF suppression (B), and their interactions on soil physicochemical properties and extracellular enzyme activity. Swc: soil water content; *R_nit_*: net nitrification rate; *R_amm_*: net ammonification rate; *R_min_*: net mineralization rate; Ure: urease; Pro: protease; Inv: Invertase; NAG: β-N-acetylglucosaminidase; BG: β-glucosidase; Cx: carboxymethyl cellulose; ACP, acid phosphatase; AKP, alkaline phosphatase. *, **, and *** indicate differences at 0.05, 0.01, and 0.001 probability levels, respectively.

Factor	Between Subjects			Within Subjects			
	B	DCD	B * DCD	T	DCD * T	B * T	B * DCD * T
Swc	1.313	0.082	0.049	33.005 ***	0.327	0.572	0.130
pH	3.816 *	10.966	1.995	23.685 ***	22.234	0.395	0.054
NO_3_^−^	0.307	3.760	0.001	112.154 ***	3.860 *	3.907 *	0.600
NH_4_^+^	3.681	386.110 ***	27.370 ***	43.989 ***	29.643 ***	3.686 *	3.400 *
*R_nit_*	31.070 ***	29.491 ***	1.563	115.186 ***	32.921 ***	4.951 **	0.616
*R_amm_*	1.561	72.660 ***	18.080 **	129.548 ***	18.875 ***	4.703 **	4.311 **
*R_min_*	0.997	10.315 *	7.789 *	53.186 ***	5.076 **	11.271 ***	2.793
Ure	0.056	0.4031	1.093	227.635 ***	1.099	0.259	1.251
Pro	3.891	1.197	14.48	55.695 ***	0.987	2.025	1.740
Inv	1.105	4.104	1.617	26.611 ***	2.156	0.394	2.482
NAG	7.864 *	0.042	0.747	4.161 *	0.313	0.841	2.102
BG	3.347	5.807 *	0.598	14.738 ***	11.735 ***	5.922 **	1.162
Cx	1.602	3.133	0.610	22.081 ***	0.866	0.183	2.382
ACP	0.750	22.400 **	6.138 *	372.874 ***	13.051 ***	1.619	4.178 *
ALP	7.081 *	7.221 *	0.996	315.834 ***	0.675	3.496 *	1.637

## Data Availability

The original contributions presented in the study are included in the article/Supplementary Materials, further inquiries can be directed to the corresponding authors.

## Supplementary Materials

The following supporting information can be downloaded at https://www.mdpi.com/article/10.3390/microorganisms13010151/s1: Figure S1: The AMF colonization in the *C. camphora* forest under different conditions after one year of the decomposition; Figure S2: Dynamics of mass remaining and soil physicochemical properties during one year of litter decomposition under different conditions; Figure S3: Dynamics of extracellular enzyme activities during one year of litter decomposition under different conditions; Figure S4: Dynamics of relative peak area of amide I, amide II, and amide III during one year of litter decomposition under different conditions; Figure S5: Spearman correlation heatmap of mass loss, soil physicochemical properties and microbial activity at different decomposition stages; Figure S6: Principal component analysis (PCA) of mass loss, soil physicochemical properties, and microbial activity at different decomposition stages; Table S1: Assay procedures of the extracellular enzymatic activities; Table S2: Olson negative exponential decay model of *C. camphora* litter decomposition. References [92,93,94,95,96,97] are cited in the supplementary materials.
