# Supplementary material for: Influence of Arbuscular Mycorrhizal Fungi on Nitrogen Dynamics During Cinnamomum camphora Litter Decomposition
Source: Microorganisms. 2025 Jan 13;13(1):151. doi: 10.3390/microorganisms13010151 (PMC11768061; doi:10.3390/microorganisms13010151)
Supplement: Supplementary file 1 [file microorganisms-13-00151-s001.zip › microorganisms-3378899-supplementary.pdf]

Supplementary materials for

Influence of Arbuscular Mycorrhizal Fungi on Nitrogen Dynamics During *Cinnamomum camphora* Litter Decomposition

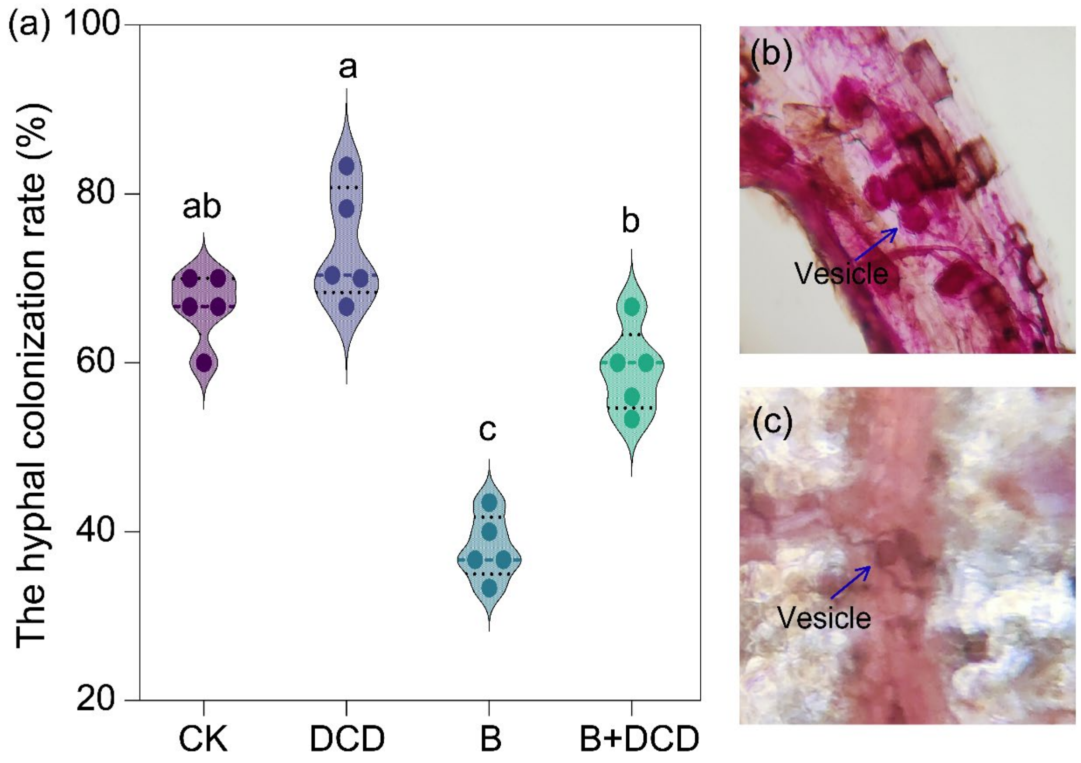

Figure S1. The AMF colonization in the *C. camphora* forest under different conditions after one year of the decomposition. The root colonization percentage (a) and AMF vesicles were observed in roots (b) that beneath the litterbags and decomposing litter (c). The violin shows the value distribution, with median value indicated by the dotted horizontal line, and different letters indicate significant difference among treatments at $p < 0.05$ level.

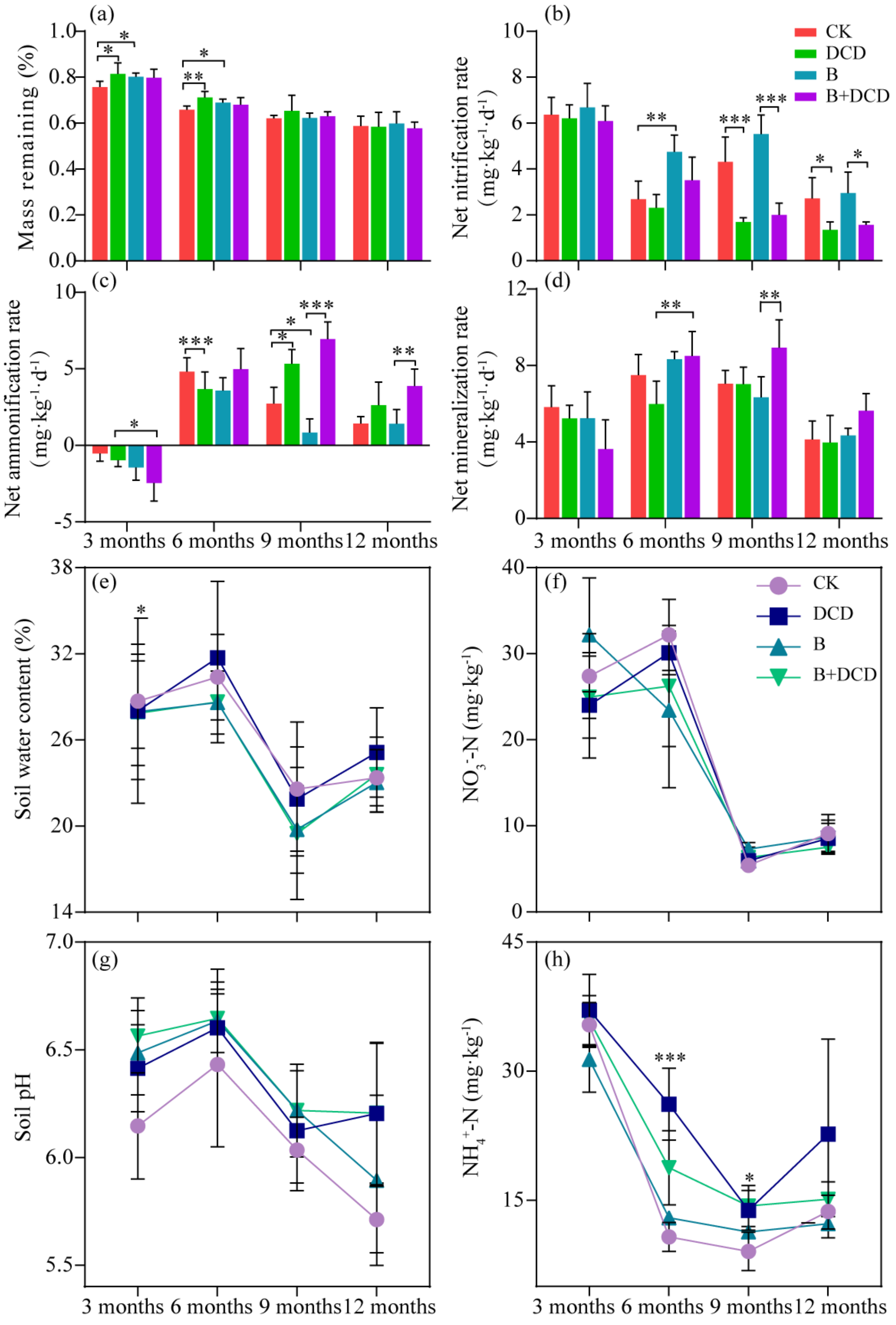

Figure S2. Dynamics of mass remaining and soil physicochemical properties during one year of litter decomposition under different conditions. (a) indicates the mass remaining, (b)-(d) indicate the net ammonification, nitrification, and mineralization rates, respectively, and (e)-(h) indicate the soil water content, NO_3^--N content, pH value and NH_4^+-N content, respectively. Values are means and standard

deviation (n = 5); *, ** and *** indicate differences at 0.05, 0.01 and 0.001 levels, respectively.

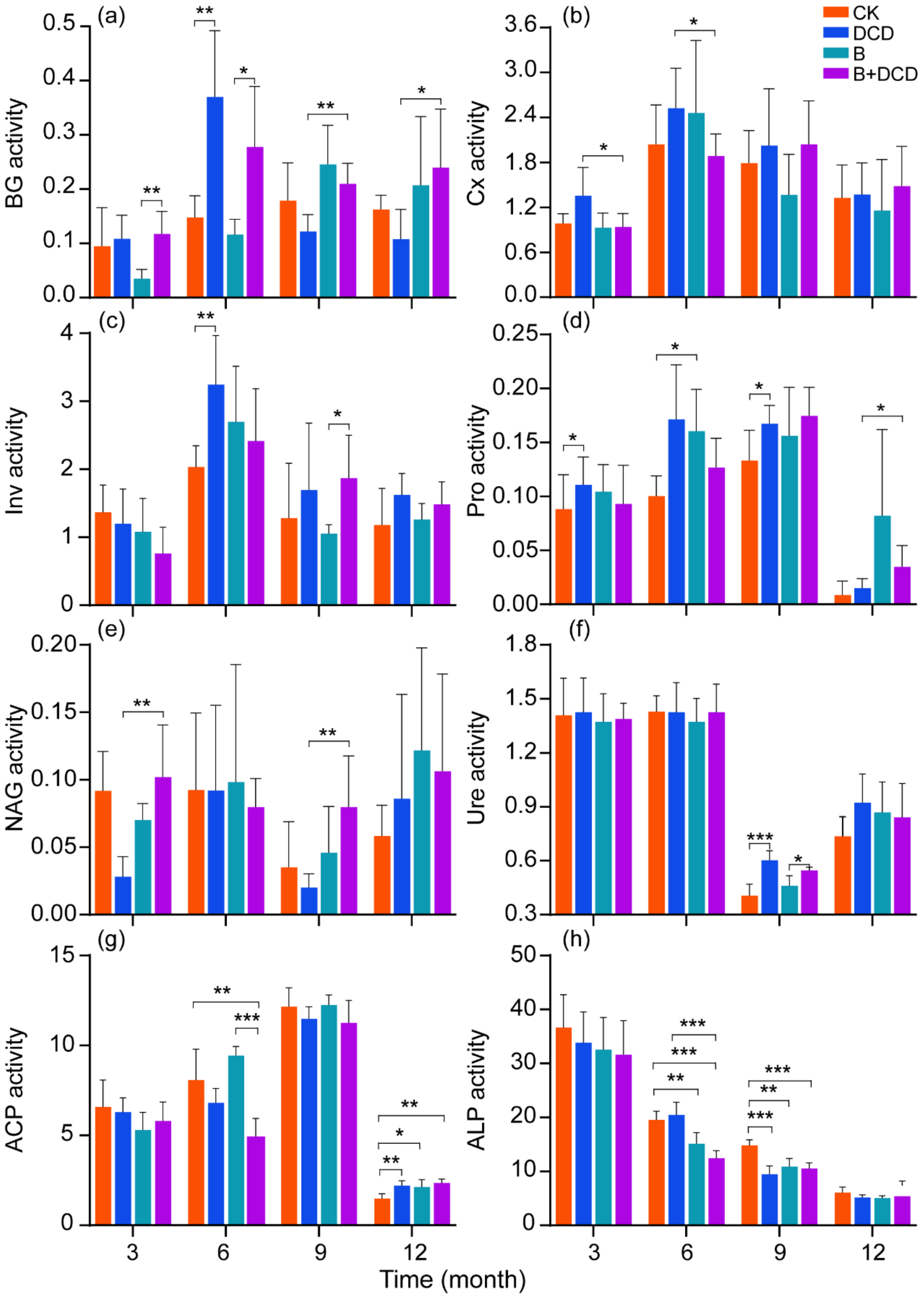

Figure S3. Dynamics of extracellular enzyme activities during one year of litter decomposition under different conditions. (a)-(h) indicate the activities of β-1,4-glucosidase (BG), carboxymethyl cellulase (Cx), invertase (Inv), protease (Pro), β-N-acetylglucosaminidase (NAG), urease (Ure), acid phosphatase (ACP) and alkaline phosphatase (AKP), respectively. Values are mean and standard deviation (n = 5); *,

** and *** indicate differences at 0.05, 0.01 and 0.001 levels, respectively. The abbreviations are the same with these in Table 2.

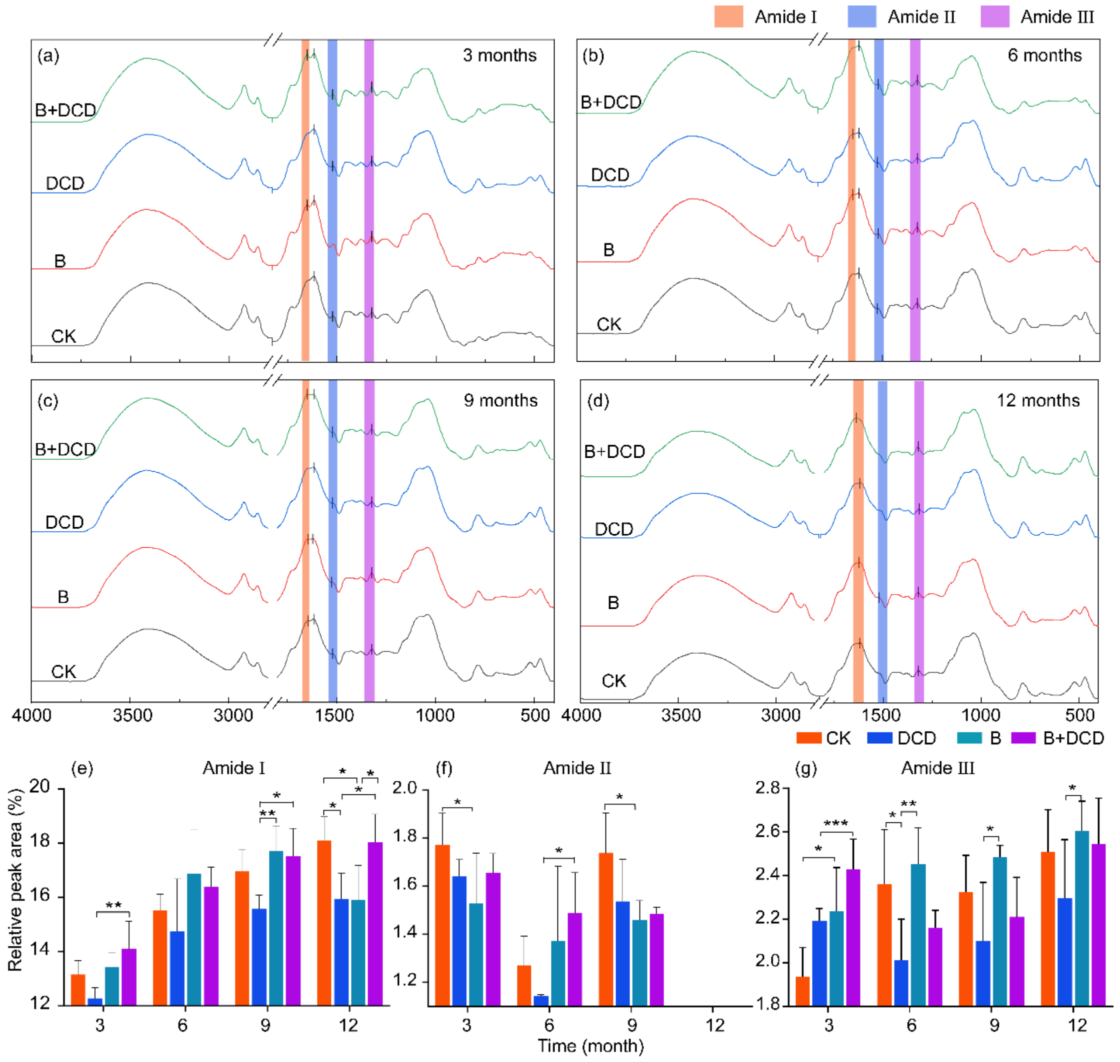

Figure S4. Dynamics of relative peak area of the amide I, amide II, and amide III during one year of litter decomposition under different conditions. (a)-(d) indicate the infrared spectral characteristics of the decomposed litter under different conditions after 3, 6, 9, and 12 months of decomposition, respectively, and (e)-(g) indicate the relative peak area of the amide I, II, and III bands, respectively. Values are mean and standard deviation (n = 5); *, ** and *** indicate differences at 0.05, 0.01 and 0.001 levels, respectively.

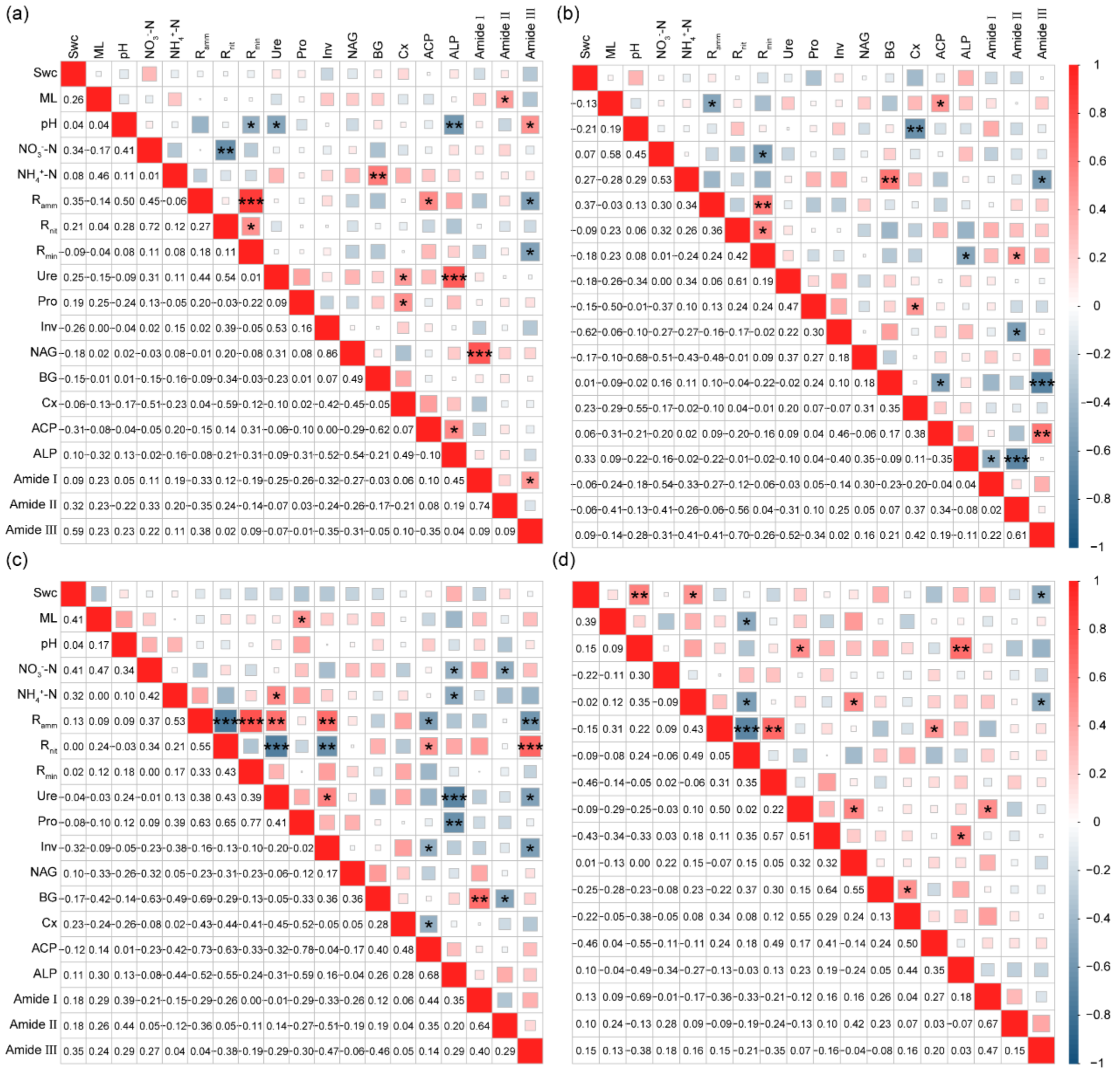

Figure S5. Spearman correlation heatmap of mass loss, soil physicochemical properties and microbial activity at different decomposition stages. (a)-(d) indicate 3, 6, 9, and 12 months, respectively, during the decomposition process. The abbreviations are the same with these in Table 2.

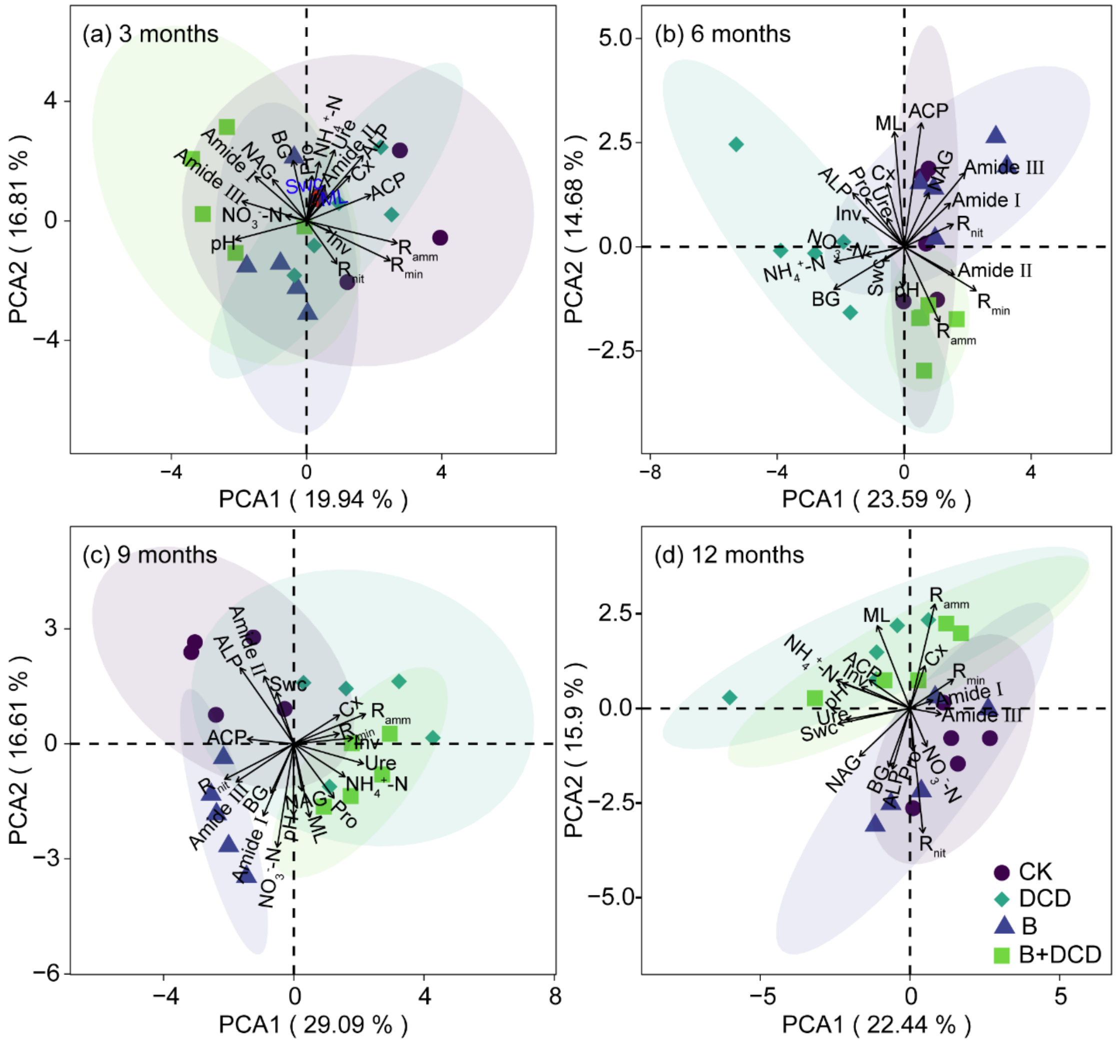

Figure S6. Principal component analysis (PCA) of mass loss, soil physicochemical properties and microbial activity at different decomposition stages. (a)-(d) indicate 3, 6, 9, and 12 months, respectively, during the decomposition process. The abbreviations are the same with these in Table 2.

Table S1. Assay procedures of the extracellular enzymatic activities.

Enzymatic	Method	IU Definition
Cellulase (E.C. 3.2.1.4)	Cellulase activity was determined using 1% carboxy-methylcellulose solution as substrate with incubation at 50 °C under pH 5.5 for 30 min; glucose concentration was determined with a spectrophotometer at 540 nm (Ghose 1987).	1 mg glucose released min^{-1} g^{-1} soil
Invertase (EC.3.2.1. 26)	Invertase activity was determined by the 3,5-dinitrosalicylic acid colorimetric method, which was expressed as the number of milligrams of glucose produced in 1 g of dry soil after 24 h (Liu et al. 2020)	1 mg glucose released h^{-1} g^{-1} soil
β-1,4-glucosidase (E.C. 3.2.1.21)	β-1,4-glucosidase activity was determined using 1.2 mM 4-nitrophenyl-β-d-linked (PNPX) glucopyranoside with incubation in the dark at 40 °C for 1.5 h (pH 5.0; 0.2 M Na_2CO_3 was used to stop the reaction), 4-Nitrophenyl (PNP) concentrations were quantified by measuring absorbance at 400 nm using a microplate spectrophotometer (Tecan Safire2, Switzerland) in 96-well plates (Vepsalainen et al. 2001).	μ mol PNP h^{-1} g^{-1} soil
Protease activity (EC 3.4)	Protease activity was determined according to Kandeler et al. (1999). Soil samples were incubated for 24 h in a buffered casein solution (pH 8.1) at 50 ℃. The aromatic amino acids released were extracted with trichloroacetic acid (0.92 M) and measured colorimetrically after adding the Folin-Ciocalteu reagent.	mg tyrosine h^{-1} g^{-1} soil
β-N-acetylglucosaminidase (EC 3.2.1.52)	β-N-acetylglucosaminidase activity was determined using *p*-nitrophenyl-N-acetyl-β-D-glucosaminide as substrate, the released *p*-nitrophenol was determined with a spectrophotometer at a wavelength of 405 nm (Urbanová et al. 2014).	μmol *p*-nitrophenol h^{-1} g^{-1} soil
Urease activity (E.C. 3.5.1.5)	Urease activity was determined using 10% urea solution as substrate with incubation at 37 ℃ for 24 h under pH 6.7; NH_4^+-N concentration was determined with a spectrophotometer at a wavelength of 578 nm (Nannipieri et al. 1980).	mg NH_3-N h^{-1} g^{-1} soil
Acid phosphatase (E.C. 3. 1.3.2) and alkaline phosphatase (E.C. 3.1.3.1)	Acid phosphatase and alkaline phosphatase activities were determined using 0.5% disodium phenyl phosphate solution as substrate with incubation at 37 °C for 24 h (pH 5.0 for acid phosphatase; pH 10.0 for alkaline phosphatase), phenol concentration was determined with the spectrophotometer at 570 nm) (Kandeler et al. 1999).	mg phenol h^{-1} g^{-1} soil

Table S2. Olson negative exponential decay model of *C. camphora* litter decomposition. *k* indicates decomposition rate, and $t_{0.5}$ and $t_{0.95}$ indicate the time (month) required for decomposition of 50% and 95% of litter mass, respectively.

Treatments	*k*	*p*	$t_{0.5}$	$t_{0.95}$
CK	0.055 ± 0.007	<0.001	12.65	54.69
B	0.051 ± 0.005	<0.001	13.51	58.38
DCD	0.049 ± 0.003	<0.001	14.08	60.84
B+DCD	0.052±0.003	<0.001	13.23	57.16

References

Ghose TK (1987) Measurement of cellulase activities. Pure and Applied Chemistry 59: 257-268. doi: doi:10.1351/pac198759020257.

Kandeler E, Tscherko D, Spiegel H (1999) Long-term monitoring of microbial biomass, N mineralisation and enzyme activities of a Chernozem under different tillage management. Biology and Fertility of Soils 28: 343-351. doi: 10.1007/s003740050502.

Liu L, Jin L, Guo Q (2020) Effects of Soil Microbiomes and Enzymatic Activities on Glechoma longituba. HortScience horts 55: 515-521. doi: 10.21273/HORTSCI14659-19.

Nannipieri P, Ceccanti B, Cervelli S, Matarese E (1980) Extraction of Phosphatase, Urease, Proteases, Organic Carbon, and Nitrogen from Soil. Soil Science Society of America Journal 44: 1011-1016. doi: https://doi.org/10.2136/sssaj1980.03615995004400050028x.

Urbanová M, Šnajdr J, Brabcová V, Merhautová V, Dobiášová P, Cajthaml T, Vaněk D, Frouz J, Šantrůčková H, Baldrian P (2014) Litter decomposition along a primary post-mining chronosequence. Biology and Fertility of Soils 50: 827-837. doi: 10.1007/s00374-014-0905-z.

Vepsalainen M, Kukkonen S, Vestberg M, Sirvio H, Niemi RM (2001) Application of soil enzyme activity test kit in a field experiment. Soil Biol Biochem 33: 1665-1672. doi: Doi 10.1016/S0038-0717(01)00087-6.
